# Timed Somatic Deletion of p53 in Mice Reveals Age-Associated Differences in Tumor Progression

**DOI:** 10.1371/journal.pone.0006654

**Published:** 2009-08-14

**Authors:** George Hinkal, Neha Parikh, Lawrence A. Donehower

**Affiliations:** 1 Department of Molecular Virology and Microbiology, Baylor College of Medicine, Houston, Texas, United States of America; 2 Interdepartmental Program of Cell and Molecular Biology, Baylor College of Medicine, Houston, Texas, United States of America; 3 Department of Molecular and Cellular Biology, Baylor College of Medicine, Houston, Texas, United States of America; 4 Department of Pediatrics, Baylor College of Medicine, Houston, Texas, United States of America; Fred Hutchinson Cancer Research Center, United States of America

## Abstract

Inactivating mutations in the p53 tumor suppressor gene occur often in the progression of human cancers. p53 inhibits the outgrowth of nascent cancer cells through anti-proliferative actions (including induction of apoptosis or senescence). To test p53 tumor suppressor functions in a novel experimental context, we somatically deleted both p53 alleles in multiple tissues of mice at various ages. Mice homozygously deleted for p53 at 3 months of age showed a longer tumor latency compared to mice deleted for p53 at 6 and 12 months of age. These results are consistent with a model in which tissues accumulate oncogenically activated cells with age and these are held in check by wildtype p53. We also deleted p53 before, concurrent with, and after treatment of mice with ionizing radiation (IR). The absence or presence of p53 during IR treatment had no effect on radiation-induced lymphoma latency, confirming that the immediate p53 damage response was irrelevant for cancer prevention. Even the presence of wildtype p53 for up to four weeks post-IR provided no protection against early lymphoma incidence, indicating that long term maintenance of functional p53 is critical for preventing the emergence of a cancer. These experiments indicate that sustained p53 anti-oncogenic function acts as a final or near final line of defense preventing progression of oncogenically activated cells to malignant tumors.

## Introduction

Genetic, epigenetic, or functional inactivation of multiple tumor suppressor genes is a hallmark of human cancers [1]. Among the most important of the tumor suppressors is p53. The p53 gene is mutated or lost in roughly half of all human cancers, and functional inactivation of p53 signaling pathways occurs in many cancers where the p53 gene remains structurally intact [2], [3]. In response to mutagenic or oncogenic stress, p53 can induce a transient or permanent cell cycle arrest or it can induce apoptosis [2]. These anti-proliferative responses of p53 prevent the emergence of a nascent cancer cell that has been activated through aberrant oncogene signaling. Oncogenic stress may activate p53 through ARF signaling or through DNA damage signaling pathways. In the traditional colorectal multi-step cancer progression model of Kinzler and Vogelstein, mutation and loss of p53 function often occurs in the transition from the adenoma stage to the malignant carcinoma stage, leading to the categorization of p53 as a “progression gatekeeper” [1], [4]. In human cancers, p53 mutation or inactivation can be an early or late event, though it is unlikely to be an initiating event in most cancers and usually occurs during cancer evolution or progression [1], [5], [6].

Mutational studies on human tumors have been very useful in defining which tumor suppressor genes are important in particular human cancer types. However, mechanistic studies of tumor suppressor function have been facilitated by the development of genetically engineered mice with tumor suppressor gene alterations. We and others have shown that mouse cancer models with germline p53 deficiency develop many types of tumors much earlier in their lifespans compared to their p53 competent counterparts [7]–[9]. Numerous new mechanistic insights into p53 tumor suppressor functions have been obtained by exploiting these mouse models [10], [11]. More sophisticated methods of manipulating the mouse genome have led to models that allow spatial and temporal regulation of gene expression [12], [13]. Some of the newer models allow somatic deletion or activation of p53 in specific tissues. Other models, discussed below, allow global activation or suppression of p53. The ability to inducibly turn p53 activity on or off provides a unique experimental flexibility in which p53 tumor suppressor function can be studied in short defined windows of time. An example is a recent paper by the Evan laboratory, which addressed the mechanisms of p53 tumor suppression function in an illuminating way. Christophorou *et al*. developed mice containing an inducible wildtype p53 allele on a p53 null background [14]. When wildtype p53 was transiently induced concurrent with ionizing radiation treatment, lymphoid and intestinal tissues were observed to undergo massive p53-induced apoptosis. However, no protection from subsequent radiation-induced lymphomagenesis was observed when compared to irradiated, non-induced mice, indicating the DNA damage response was dispensable for long-term prevention of tumors due to acute stress. However, if wildtype p53 was transiently induced 8 days post-irradiation, circumventing the acute damage response and instead allowing mutations to develop, significant protection from radiation-associated lymphomas was noted as these mice survived 50% longer. This protection was dependent on ARF, a protein that activates p53 in response to oncogenic stress [15]. Thus, the p53-mediated arrest response to oncogenic activation (even if short term) is critical for protection from cancer [14]. This study has been important in shifting our focus from the immediate DNA damage response mechanisms of p53 to the ARF-mediated oncogenic response of p53 when considering its primary cancer suppressive role.

In this paper, we attempted to address aspects of p53 tumor suppressor function through a novel approach. Instead of generating p53 germline mutations, we generated mice that allow global somatic deletion of one or both copies p53 at any chosen time. We homozygously deleted p53 at various time points before, concurrent with, and up to four weeks after ionizing radiation and monitored for radiation-induced lymphomas. Surprisingly, all groups of irradiated mice exhibited roughly the same median time to lymphoma formation after deletion of p53 (and much sooner than p53 deleted non-irradiated controls). Thus, even retention of p53 for up to 4 weeks post-damage may confer little or no protection from early lymphomagenesis. Instead, a sustained p53 anti-oncogenic response is required. We also examined the kinetics of spontaneous tumorigenesis in mice deleted for both p53 alleles at different ages and compared these to mice with germline deletions in both p53 alleles. As expected, we found that somatic p53 deletion delayed onset of tumors compared to those mice with germline p53 deletions, consistent with the age when p53 was deleted. However, when median tumor latencies were adjusted for time post-deletion, mice deleted for p53 at 3 months of age showed a delay in tumorigenesis relative to mice deleted for p53 at 6 and 12 months of age. We conclude that oncogenically activated cells accumulate rapidly with age in a p53-independent fashion and that wildtype p53 likely acts as a critical final or near final line of defense in suppressing these oncogenically activated clones.

## Results

### Somatic deletion of p53 in mouse tissues

**Figure 1:**
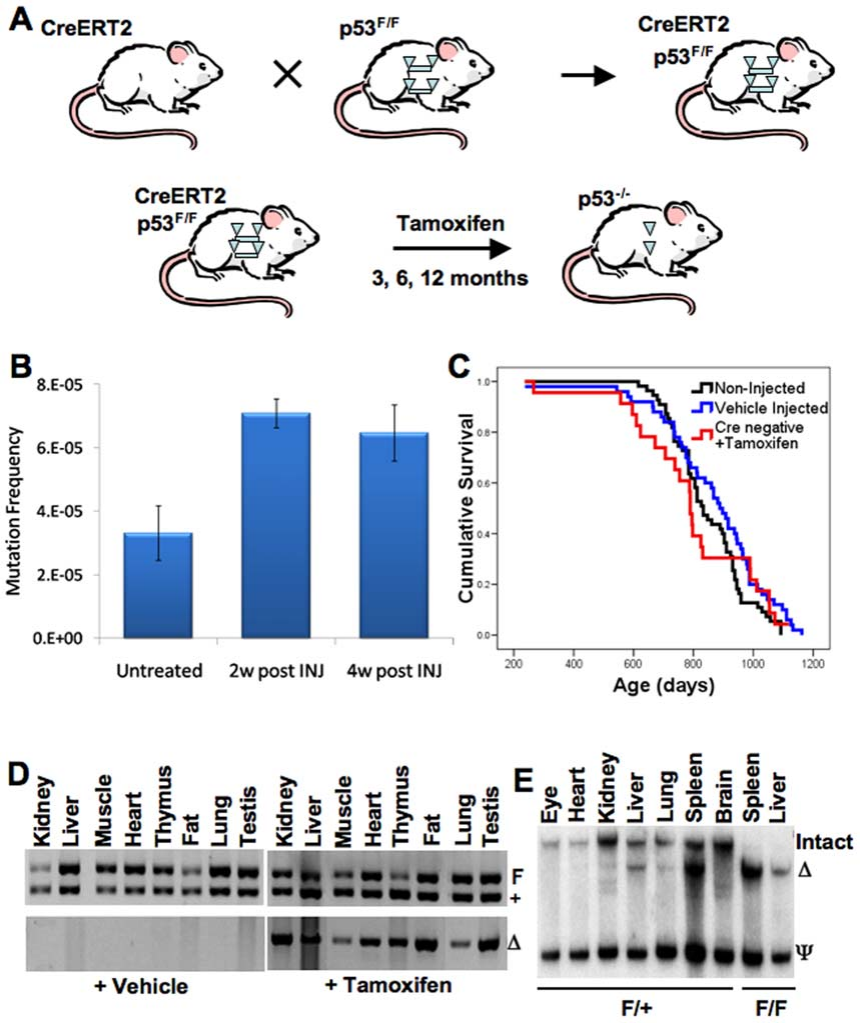
CreERT2-p53^F/F^ and CreERT2^+/F^ mice display efficient p53 allele excision in many tissues after tamoxifen treatment. (A) Experimental design to generate mice that can inducibly and somatically delete p53 in many tissues. Floxed p53 alleles are represented by triangle flanked bars. Cre-excised p53 alleles are indicated by solo triangles. (B) Tamoxifen treatment of wildtype mice moderately elevates liver DNA mutation frequency. Three month C57BL/6 Big Blue Mice designed to measure mutation frequencies were treated with tamoxifen (five 1 mg daily injections) and sacrificed at 2 or 4 weeks post-injection and liver DNA subjected to the mutation frequency assay as described in the methods. Mutation frequencies are shown with or without tamoxifen injection (n = 3 for each time point, ±standard error of the mean). (C) Tamoxifen injection of mice has little or no effect on survival compared to vehicle-injected wildtype mice. Tamoxifen injection of CreERT2 negative p53^F/F^ and p53^+/F^ mice (red curve, n = 23) showed similar Kaplan-Meier survival curves as wildtype uninjected mice (black curve, n = 55) and vehicle injected CreERT2-p53^+/F^ mice (blue curve, n = 50). (D) PCR assays show that vehicle-injected CreERT2-p53^+/F^ tissues exhibit no p53 allele excision, while all tamoxifen-injected CreERT2-p53^+/F^ tissues show evidence of p53 allele excision. Upper panels show genotyping PCR where the upper band (F) is the larger non-excised floxed allele of p53 and the lower band (+) is the non-floxed wild type p53 allele from various CreERT2-p53^+/F^ tissues. The lower panels show PCR fragments specific for the excised p53 allele (Δ). The left set of panels contain results from vehicle (corn oil) treated tissues while the right set of panels contain PCR results from tamoxifen treated tissues. Note that in the absence of tamoxifen there is no background p53 allele excision and that in the presence of tamoxifen all tissues show evidence of p53 allele excision. (E) Tamoxifen treatment of CreERT2-p53^+/F^ and CreERT2-p53^F/F^ mice results in efficient p53 allele excision (Δ) in some, but not all tissues. Southern blot analysis of genomic DNA from various CreERT2-p53^+/F^ and CreERT2-p53^F/F^ tissues was performed as described in the methods. Note that spleen and liver show efficient excision, lung and kidney show partial excision, while eye and brain show little evidence of excision.

To examine the effects of global p53 gene deletion at specific ages in mouse tissues, we employed the strategy outlined in Fig. 1A. We crossed two genetically engineered mice: “floxed” p53 mice and Rosa26-CreERT2 knock-in mice (Fig. 1A). Floxed p53 mice carry an intact wildtype p53 allele containing two loxP recombination sites (p53^F/F^) in introns 1 and 10 of p53 [16]. CreERT2 mice contain a knock-in, modified Cre recombinase ubiquitously expressed from the *Rosa26* locus [17]. The modified Cre recombinase expressed from the CreERT2 allele is fused to a mutated form of the estrogen receptor that is non-responsive to estrogen but highly sensitive to the estrogen receptor antagonist 4-hydroxytamoxifen (a downstream metabolite of tamoxifen) rendering it inactive until the addition of ligand [17]. Thus, intraperitoneal injection of the progeny CreERT2-p53^+/F^ or CreERT2-p53^F/F^ mice with tamoxifen results in activation of the Cre recombinase and deletion of one (p53^+/F^) or both (p53^F/F^) p53 alleles spanning exons 2 through 10 in multiple tissues.

The use of tamoxifen as an inducer raised the issue of whether it could have p53-independent effects on tumorigenesis, since some studies had shown tamoxifen-induced pathology and DNA adduct formation in rodent livers [18]. To ascertain whether tamoxifen injection itself was mutagenic or carcinogenic in mice, we injected 3 month old Stratagene Big Blue transgenic mice with our standard tamoxifen injection regimen (five consecutive daily intraperitoneal injections of 1 mg tamoxifen in corn oil). The Big Blue mouse is a transgenic mouse that contains non-expressed bacterial selectable markers that can be used to quantify genomic mutation frequencies in well defined assays [19]. Two or four weeks after tamoxifen injection, livers were harvested from the treated mice and genomic DNA was analyzed in the Big Blue mutation assay. The resulting data indicate that tamoxifen treatment significantly increases (p_2-week_ = 0.0089, p_4-week_ = 0.032) liver mutation frequency by roughly two-fold in mice (Fig. 1B). However, we found that wildtype mice injected with tamoxifen at 3 months of age exhibit very similar survival curve dynamics (Tamoxifen treated wildtype mice versus p_vehicle_ = 0.269, p_non-injected_ = 0.572), longevities (maximal lifespan of: tamoxifen control = 1119 days, vehicle control = 1162 days) and tumor susceptibilities as vehicle injected control mice (Fig. 1C). Thus, tamoxifen injection may lead to a modest increase in liver DNA mutation frequency but it does not affect overall longevity or tumor susceptibility.

To detect whether tamoxifen treatment of CreERT2-p53^F/F^ and CreERT2-p53^+/F^ mice was efficient in global excision of p53 throughout all tissue types, we injected several of these mice at three months of age and harvested a variety of tissues two weeks later. Examination of genomic DNA from multiple tissues of the tamoxifen-treated and non-treated mice by PCR analyses showed no evidence of background p53 allele excision in non-treated mice, but detectable excision in all tissues of treated mice (Fig. 1D). To obtain a more quantitative estimate of relative allele excision in individual tissues of the CreERT2-p53^+/F^ and CreERT2-p53^F/F^ treated mice, Southern blot analysis was performed (Fig. 1E). The Southern blots showed essentially complete excision of the p53 alleles in spleen and liver and moderate levels of excision of kidney and lung, and low levels of excision in brain, heart, and eye; similar to what had been reported previously in the Rosa26-CreERT2 line [20]. We examined the kinetics of tamoxifen-mediated p53 deletion in the livers and spleens of CreERT2-p53^+/F^ and CreERT2-p53^F/F^ mice and found significant deletion after just one day of injections (data not shown). Tamoxifen treatment of 3 and 12-month old CreERT2-p53^F/F^ mice resulted in similar efficiencies of allele excision in each tissue examined, indicating that age does not affect allele excision frequency (data not shown).

### Effects of p53 allele deletion on radiation-induced lymphomagenesis

**Figure 2:**
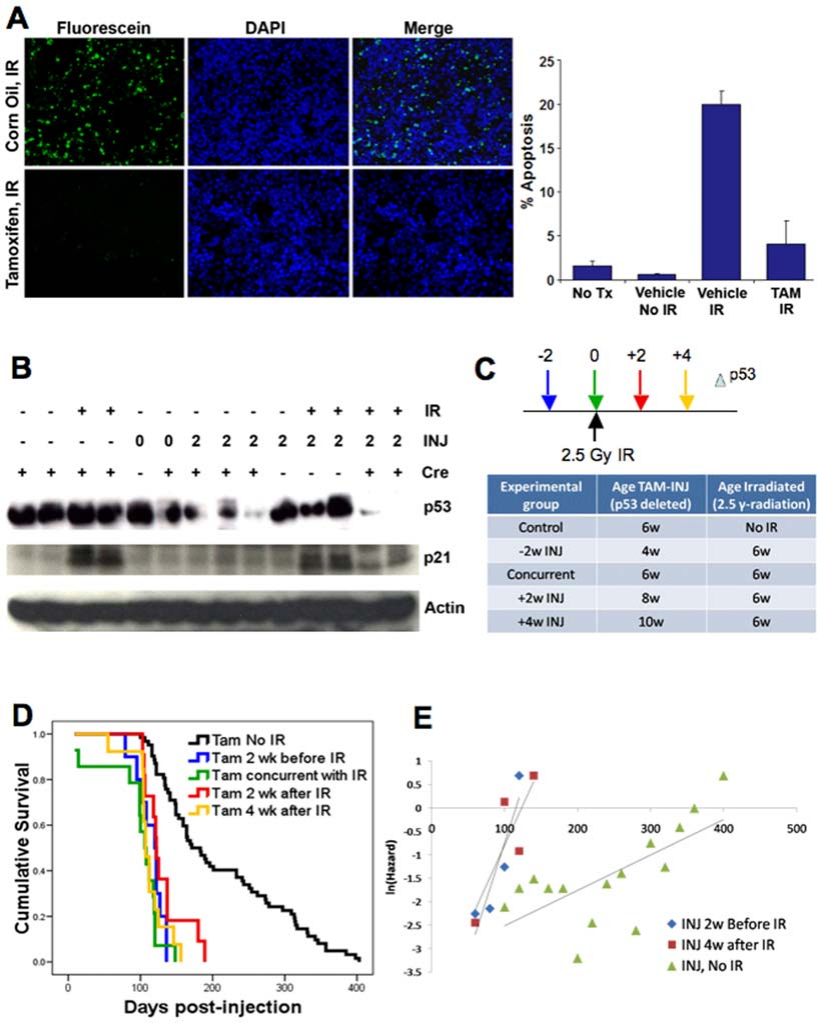
Tamoxifen treatment of CreERT2-p53^F/F^ mice before, during, and after IR demonstrates the role of p53 in suppressing carcinogen-induced tumorigenesis. (A) Deletion of p53 prior to irradiation prevents robust apoptotic response in CreERT2-p53^F/F^ mice. Three month old CreERT2-p53^F/F^ mice were injected with tamoxifen or vehicle (corn oil) two weeks prior to treatment with 5 Gray ionizing radiation. Five hours after IR treatment, spleens were harvested and tissue sections subjected to TUNEL assays for apoptotic cells. Note strong apoptotic response in vehicle treated mice and greatly reduced apoptotic response in tamoxifen-treated mice. Panel to the right shows percentage of TUNEL positive cells in spleens from untreated mice (No Tx), corn oil injected and non-irradiated mice (vehicle, No IR), corn oil injected and irradiated mice (vehicle, IR), and tamoxifen injected and irradiated mice (TAM, IR). (B) The p53 damage response is abrogated in tamoxifen injected CreERT2-p53^F/F^ mice subjected to IR. Spleens were harvested from non-irradiated (-IR) and irradiated (+IR), non-injected (-INJ) and injected (+INJ 0 or 2 weeks post-injection) CreERT2-p53^F/F^ (+Cre) and p53^F/F^ (-Cre) mice and Western blots performed on tissue lysates using p53, p21, and actin antibodies. Note that robust p53 and p21 expression indicative of a robust p53 response occurs only in irradiated but non-Cre expressing or non-injected mice. (C) Six week old CreERT2-p53^F/F^ mice were subjected to 2.5 Gy ionizing radiation and were injected with tamoxifen either 2 weeks prior (n = 10), simultaneous with (n = 14), 2 weeks after (n = 11), or 4 weeks after (n = 13) IR exposure. Non-irradiated tamoxifen treated mice CreERT2-p53^F/F^ are included as controls (n = 62). (D) Kaplan-Meier tumor-free survival curves of IR-treated CreERT2-p53^F/F^ mice show similar lymphoma latency of lymphomas post injection while non-IR treated tamoxifen-injected mice showed more delayed tumorigenesis. (E) Natural logarithm of mortality rates fitted using the T4253H smoothing algorithm comparing Gompertz variables from CreERT2-p53^F/F^ mice injected with tamoxifen at 2 weeks prior and 4 weeks after IR treatment and non-IR treated tamoxifen-treated CreERT2-p53^F/F^ mice (x-axis in days). Note that the IR-treated mice exhibit high mortality whether or not p53 is present at the time of radiation.

To examine the effects of a carcinogen on p53 tumor suppressor function, we assessed the effects of somatic p53 allele loss on radiation-induced lymphomagenesis. First, to show that p53 allele excision affects the p53-mediated apoptotic response to DNA damage, we performed TUNEL assays on tissue sections of irradiated spleens (5 hours after 5 Gy IR) from tamoxifen or vehicle-treated CreERT2-p53^F/F^ mice. Tamoxifen-injected mice showed little evidence of apoptotic cells, consistent with the deletion of p53 in these tissues (Fig. 2A). In contrast, spleen sections from vehicle-injected and irradiated CreERT2-p53^F/F^ mice exhibited high levels of apoptosis, indicating that the p53-mediated response was intact in these mice. Next, we examined p53 and p21 (CDKN1A) protein levels in the irradiated and mock-irradiated spleen tissues of tamoxifen treated and untreated CreERT2-p53^F/F^ mice 5 hours post-IR (Fig. 2B). p21 is a target gene of activated p53 that is transcriptionally induced following DNA damage. We found that p53 protein was expressed at moderate to high levels in all tissues where p53 alleles were intact and at reduced levels where the p53 alleles were excised. As expected, p21 levels were only increased in spleens of mice treated with IR and where p53 was not excised. Radiation and tamoxifen treated CreERT2-p53^F/F^ mice showed little or no increase in p21 levels, as expected (Fig. 2B).

Experiments by the Evan laboratory performed with mice containing an inducible wildtype p53 allele showed that after a carcinogenic insult, p53 could delay cancer if activated several days after the insult rather than during the insult. This suggested that the immediate p53-mediated damage response was less important than the subsequent anti-oncogenic response in preventing the emergence of tumors [14]. Here, we used the CreERT2-p53^F/F^ mice in a reverse manner. Rather than inducing p53, we deleted both p53 alleles at various time points before, during, or after 2.5 Grays of ionizing radiation (IR) treatment. This IR dose is sufficient to induce rapid lymphoma formation in mice without p53, but insufficient for early lymphomas in wildtype mice [21]. We injected the CreERT2-p53^F/F^ mice with tamoxifen 2 weeks prior (-2), during (0), 2 weeks after (+2), and 4 weeks after (+4) whole body irradiation, ten to twelve mice for each cohort. Tamoxifen injected non-irradiated CreERT2-p53^F/F^ mice were used as controls. Surprisingly, when adjusted for time post-tamoxifen injection, all four tamoxifen-treated CreERT2-p53^F/F^ groups exhibited roughly the same median time to lymphoma formation and survival curves (p_−2:0_ = 0.140, p_−2:+2_ = 0.211, p_−2:+4_ = 0.696), considerably earlier than the control mice in which wildtype p53 was deleted, but without IR treatment (Fig. 2C,D). The steepness of the post-irradiation survival curves in Fig. 2D indicated very high mortality rates in tamoxifen treated and irradiated CreERT2-p53^F/F^ mice compared to the tamoxifen-treated, non-irradiated CreERT2-p53^F/F^ mice. Such high mortality rates were confirmed in Gompertz variable analyses shown in Fig. 2E, where only modest mortality rate differences were observed between CreERT2-p53^F/F^ mice deleted for p53 2 weeks prior to or 4 weeks after IR treatment (p = 0.023). Both of these rates were dramatically higher than those for tamoxifen treated but non-irradiated CreERT2-p53^F/F^ mice (p = 0.0016 and p<0.0001) (Fig. 2E). Thus, even if wildtype p53 is retained for up to four weeks after radiation, the time to lymphomagenesis is essentially the same as if p53 was absent during radiation. These results confirm the earlier data from Christophorou *et al.* [14] that an intact and immediate p53 DNA damage response is irrelevant to subsequent lymphoma susceptibility. However, our data indicate retention of p53 for even up to 4 weeks post-IR treatment may confer little or no protection from early lymphomagenesis. Thus, it seems that a sustained p53 anti-oncogenic response for months after the initial damage is required for full protection against early lymphomas.

### Spontaneous tumorigenesis in mice with homozygous p53 allele deletion at 3, 6, and 12 months of age

**Figure 3:**
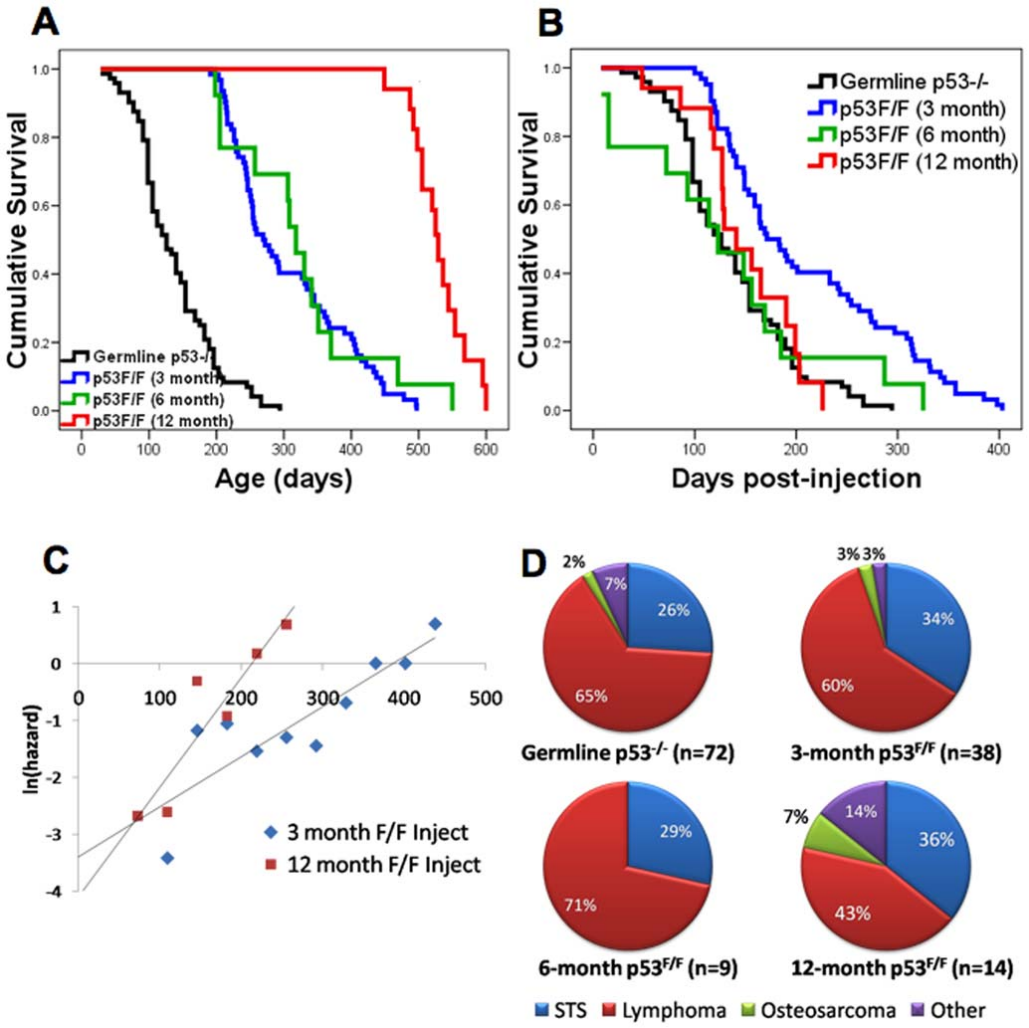
Tumor-free survival curves of CreERT2-p53^F/F^ mice injected with tamoxifen at 3, 6, and 12 months of age. (A) Kaplan-Meier survival curves are shown for germline p53^−/−^ mice in black (n = 72), CreERT2-p53^F/F^ mice injected with tamoxifen at 3 months in blue (n = 62), at 6 months in green (n = 13), and at 12 months in red (n = 18). (B) Tumor-free survival curves adjusted for time after tamoxifen injection. Line colors represent the same groups as in A. Note that the CreERT2-p53^F/F^ mice injected with tamoxifen at 6 and 12 months of age exhibit a similar median survival as p53^−/−^ mice and a reduced median survival compared to their 3 month injected counterparts. (C) Natural logarithm of mortality rates fitted using the T4253H smoothing algorithm comparing Gompertz variables from CreERT2-p53^F/F^ mice injected with tamoxifen at 3, 6 and 12 months (x-axis in days) show a significantly increased mortality rate in the 12 month injected mice (p = 0.0007). (D) Tumor types observed in CreERT2-p53^F/F^ mice treated with tamoxifen at 3 and 6 months are similar to those observed in germline p53^−/−^ mice. In 12 month injected CreERT2-p53^F/F^ mice, carcinomas show a modest increase and lymphomas a modest decrease.

To determine the effects of losing both p53 alleles at various ages, we tamoxifen treated CreERT2-p53^F/F^ mice at 3, 6, and 12 months of age. These mice and control tamoxifen-treated p53^F/F^ mice without the CreERT2 allele were then monitored over their lifespan and examined for kinetics of tumor incidence. Tumor-free survival curves of CreERT2-p53^F/F^ mice injected with tamoxifen at 3, 6, and 12 months were compared with germline p53^−/−^ mice (n = 62, 13, 18, and 72 respectively). As expected, tumors arise later in tamoxifen-treated CreERT2-p53^F/F^ mice compared to p53^−/−^ mice (Fig. 3A). The 3 month tamoxifen-injected CreERT2-p53^F/F^ mice showed a median tumor incidence about 140 days later than the p53^−/−^ mice and the two survival curves paralleled each other very closely. Surprisingly, the survival curve of the 6 month tamoxifen treated CreERT2-p53^F/F^ mice overlapped the 3 month cohort (p = 0.598), and developed tumors at nearly the same mean age as the 3 month old floxed p53 mice (305 days for the 3 month cohort, 323 days for the 6 month group). The 12 month old tamoxifen-treated CreERT2-p53^F/F^ mice developed tumors with a median incidence of 529 days. When we adjusted tumor-free survival (Fig. 3B) to the time of tamoxifen injection, it was clear that later excision of p53 (6 and 12 months) resulted in a reduced median tumor latency relative to excision of p53 at 3 months (p_3:6_ = 0.005, p_3:12_ = 0.014, p_6:12_ = 0.389). Moreover, the tumor-free survival curves increased in “steepness” in the later tamoxifen treated CreERT2-p53^F/F^ mice, again suggesting an increase in mortality rates in the older treated mice. Comparison between the 3 month and 12 month treated CreERT2-p53^F/F^ Gompertz variables confirmed increasing mortality rates with later injection (p = 0.0007, Fig. 3C). Thus, tumors in the later injected CreERT2-p53^F/F^ cohorts exhibit a reduction in median and maximum tumor-free post-injection survival, suggesting that these mice have an increased accumulation of pre-neoplastic or neoplastic clones that require loss of p53 to allow full tumor progression.

When we performed necropsies and histopathology on the tumors of the various tamoxifen treated CreERT2-p53^F/F^ mice, we found that 3 and 6 month treated mice developed primarily lymphomas and secondarily soft tissue sarcomas, at ratios of roughly 2-3:1, similar to that of p53^−/−^ mice (Fig. 3D). In contrast, lymphomas in the 12 month treated CreERT2-p53^F/F^ mice comprised only 43% of total tumors, and there were more soft tissue sarcomas and carcinomas (36 and 14% respectively). This is more consistent with tumor observations in p53^+/−^ mice, where retention of one wildtype p53 allele promotes later tumors which are more mixed in type [22].

## Discussion

In this paper, our goal was to better understand p53 tumor suppressor functions by employing a novel experimental approach. Rather than examining the effects of p53 germline deletion, we chose to somatically delete p53 in multiple mouse tissues at varying ages and at varying times before, during, and after a carcinogenic insult. This approach more accurately mimics the late somatic p53 loss that often occurs in human sporadic tumors (though massive simultaneous deletion of p53 is admittedly artificial). The bi-allelic progeny of the CreERT2 Rosa26 knock-in and floxed p53 knock-in mice allowed a very precise temporal regulation of p53 deletion in most tissues of the mouse. We found that efficient deletion occurred within 24 hours of the first tamoxifen injection and that the age of injection had little effect on deletion efficiencies. While the p53 gene in tissues such as brain, heart, and eyes were inefficiently excised, the tissues most susceptible to tumors in p53^−/−^ mice (lymphoid and mesenchymal tissues) were generally quite susceptible to p53 allele excision. The tumor types observed in the somatically deleted p53 mice were very similar to those observed in p53^−/−^ mice. The results presented here suggest that the incomplete p53 allele deletion in some tissues has only marginal, if any, effects on overall tumor incidence in this model.

**Figure 4:**
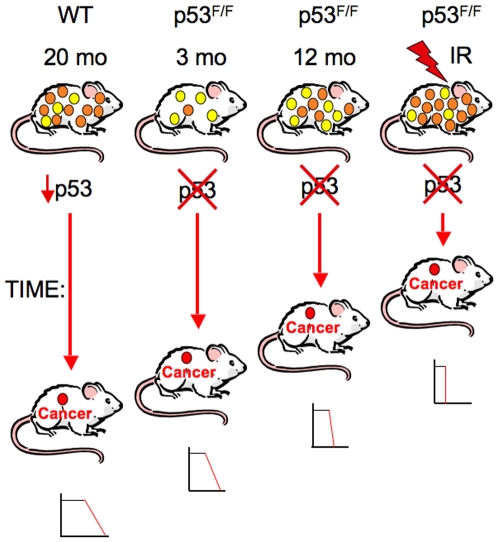
p53 acts as a gatekeeper by preventing progression of oncogenically activated cells to full malignancy. From left to right 20 month old wildtype p53 mice, p53^F/F^ mice with intact p53 until 3 and 12 months of age, and 6 week old irradiated p53^F/F^ mice are shown. All of these mice accumulate oncogenically activated clones with age, though irradiation greatly enhances the numbers of such clones. Oncogenically activated clones are represented by yellow (early stage oncogenic clones that are not yet capable of tumor formation) and orange (later stage oncogenic clones capable of tumor formation upon loss of p53) circles. These clones are held in check by wildtype p53, which prevents their progression to full malignancy. However, after the age of 20 months in wildtype mice, p53 functional activities decline, which could lead to malignant progression of one or more oncogenic clones (cancers indicated by red circle). When p53 is deleted by tamoxifen injection at 3, 6, or 12 months or following IR treatment in CreERT2-p53^F/F^ mice, some oncogenic clones will progress to form a malignant cancer. For the p53^F/F^ mice, the median and maximal tumor latencies following p53 deletion decrease with age or with carcinogenic insult. The relative tumor latencies are indicated by the length of the red vertical arrows. For the wildtype mice, since p53 activity is merely reduced, malignant progression is more delayed. The small graphs below each of the tumor bearing mice shows the shape of the survival curves for each cohort, indicating that tumor latencies and mortality rates depend on p53 functional status, age, or the presence of carcinogenic insult. Thus, p53 acts as a final or near-final gatekeeper keeping these oncogenic clones from progressing to full malignancy.

The age-specific somatic p53 deletion experiments were designed to test whether the aging process affects p53 tumor suppressor functions. When p53 was homozygously deleted at 3, 6, and 12 months of age, median tumor latencies after p53 deletion were 171, 123, and 141 days, respectively, compared to germline deleted p53^−/−^ mice with a 128 day median tumor latency. The 6 and 12 month post deletion tumor incidence curves were significantly different from those of the mice deleted for p53 at 3 months (p = 0.005 and 0.014, respectively). In addition to median tumor latencies being reduced in the older p53 deleted mice, mortality rates also accelerated with age (Fig. 3C). By these criteria, rates of tumor progression after p53 deletion appear to increase with age. As shown in the model presented in Fig. 4, we hypothesize that these effects are the result of age-associated accumulation and progression of oncogenically activated cell clones in the mice. At three months of age, few such clones have accumulated or progressed to a stage where they can form an immediate cancer once p53 is deleted. Therefore, tumor development is delayed and mortality rates are low after p53 excision. However, by 12 months, increased numbers of oncogenically activated clones have progressed and await only the inactivation of p53 to become fully malignant (Fig. 4). Based on the rapidity of post-deletion tumor latency in the older mice, it is likely that for some of these precancerous clones, oncogenic activation of wildtype p53 is the final or near-final barrier preventing conversion to a malignant tumor. When p53 is deleted, such clones transition to malignancy. Prior to p53 deletion oncogenic clones clearly accumulate and evolve in the presence of active wildtype p53, suggesting that the presence of p53 may have little or no effect on early neoplastic evolution, but its loss can certainly accelerate progression to malignancy.

One interesting result in these analyses was that the 3 month p53 deleted mice had a significant delay in median tumor incidence relative to germline p53^−/−^ mice (p = 1.24×10^−7^), whereas the 6 and 12 month homozygous deletion incidence curves were not significantly different from germline p53^−/−^ mice (p = 0.987 and 0.247, respectively). As one potential explanation for the tumor latency differences between the p53−/− and 3 month p53 deleted mice, we hypothesize that p53 has a significant protective effect on genomic integrity during embryogenesis and early postnatal development, when cell division rates are high and prone to potential chromosomal abnormalities. p53 has known roles in G2/M and mitotic spindle checkpoints and centrosome integrity and its absence would be expected to increase frequencies of chromosomal missegregation [23], [24]. Indeed, 4–6 week old p53 null mice have dramatically higher fractions of aneuploid cells in bone marrow, lymphoid and other tissues compared to normal mice [25], [26]. Thus, early in life the presence of p53 may protect against genomic instability that might facilitate formation of oncogenic clones, whereas later in life it protects against progression of oncogenic clones, as hypothesized in some models for p53 tumor suppressor function [27]–[29].

These experiments also suggest that wildtype p53 cannot eliminate all oncogenic clones through apoptosis or keep them permanently arrested through senescence. Instead, activated wildtype p53 merely holds some of these oncogenic clones in stasis, suppressing their further malignant evolution only as long as p53 remains functional. Changes in p53 function may also explain in part why tumor incidence in wildtype mice rises dramatically with age. Levine and colleagues have recently demonstrated that p53 DNA damage responses, transcriptional activities and apoptotic functions decline significantly in older mice [30]. As shown in the model in Fig. 4, reduced p53 activity in older p53 wildtype mice could enhance progression of oncogenically activated clones to malignancy, but this progression would not be nearly as rapid as in the somatically deleted p53^F/F^ mice, where p53 loss is immediate and complete. Moreover, the recently described cancer resistant transgenic and knock-in mice that express additional copies or more active versions of p53 could have more efficient suppression of oncogenically activated cell clones late into old age [31]–[34].

Agents that induce acute DNA damage are often carcinogenic. The capacity for p53 to respond to both DNA damage and aberrant oncogenic signals suggested that both activities might be important in its tumor suppressor function following a carcinogenic insult such as ionizing radiation treatment. However, Christophorou *et al.* [14] showed in recent experiments on mice expressing an inducible wildtype p53 allele that the immediate p53 DNA damage response was irrelevant to prevention of radiation-induced lymphomas. Instead, the ARF-dependent p53 response to oncogenic activation was critical for delaying lymphomagenesis. Here, rather than activating p53 following treatment of mice with ionizing radiation, we deleted p53 at various time points before, concurrent with, and after irradiation. In many ways, our results confirm those of the Evan laboratory [14]. Whether or not wildtype p53 was present at the time of radiation treatment had no effect on subsequent lymphoma incidence. Mice in which p53 was deleted before, during, or after IR treatment displayed the same lymphoma latency. Surprisingly, however, we found that retention of wildtype p53 for as long as four weeks post IR treatment conferred no additional protection from lymphoma than mice that were deleted for p53 two weeks prior to radiation. Mice in which p53 was deleted four weeks after IR treatment showed the same median lymphoma latency and mortality rate as did mice in which p53 was deleted two weeks before irradiation (p = 0.696), relative to when p53 was deleted. This indicates that sustained wildtype p53 function is critical for suppressing tumorigenesis and that p53 may only keep some oncogenically activated cells in check rather than permanently disable or destroy them. This absence of wildtype p53 protective effect for up to four weeks post-IR that we observed differed from that observed by the Evan group, who showed that even a short six day activation of wildtype p53 eight days post-IR resulted in a significant delay in tumorigenesis. The reasons for the discrepancies are unclear, though they may be due to different features of the two models or that the Evan p53-ER fusion protein was a more robust tumor suppressor being activated in a cellular environment previously missing p53 entirely.

These studies support a model in which p53 acts as a “progression gatekeeper” preventing oncogenically activated cell clones from becoming fully malignant. Such cell clones progress and increase in numbers with age and p53 may act as a final or near final line of defense against cancer. It is only with old age that p53 anti-cancer function may decline and cancers may finally emerge (Fig. 4). We have also confirmed that the acute DNA damage response of p53 is likely to be unimportant and that a long term continuous p53 response to oncogenically activated cells is likely to be critical for cancer suppression. While p53 may prevent cancer progression by inducing apoptosis or senescence in some oncogenically activated cells, for other such clones it merely prevents further malignant evolution. The somatic p53 deletion model presented here has been a source of useful insights into p53 tumor suppressor function and it may provide additional insights through further experimental manipulations.

## Materials and Methods

### Mice

#### Ethics Statement

All animals were handled in strict accordance with good animal practice as defined by the Institutional Animal Care and Use Committee for Baylor College of Medicine and Affiliates (Animal Protocol AN336) and by the Association for the Assessment and Accreditation of Laboratory Animal Care (AAALAC), Guide for the Care and Use of laboratory Animals (NRC1996).

FVB.129 doubly floxed (p53^F/F^) mice were obtained from the National Cancer Institute Mouse Models of Human Cancers Consortium and were originally generated by Anton Berns as previously described [16]. These mice were backcrossed into a C57BL/6 background. C57BL/6.SLJ CreERT2 homozygous mice were obtained from Artemis Pharmaceuticals GmbH [17] and backcrossed into a C57BL/6 background. C57BL/6 Big Blue mice were obtained from Stratagene (Austin, TX). All mice were bred and maintained in a specific pathogen-free animal facility at Baylor College of Medicine. CreERT2-p53^F/F^ mice were generated by successive crossing of CreERT2 and p53^F/F^ mice and genotyping by PCR as described below. All research with mice has been conducted in compliance with the Baylor Animal Protocol Committee (Baylor College of Medicine Animal Protocol AN336) and AAALAC recommendations as published in the The *Guide for the Care and Use of Laboratory Animals* (NRC1996).

#### Allele genotyping

High molecular weight genomic DNA was prepared from 5 mm tail tips or 50 mg tissue and prepared as previously described [35]. All PCR primers were synthesized by Integrated DNA Technologies (Coralville, IA). The presence of the CreERT2 gene was verified using the primers Cre-F: 5′-AAG AAC CTG ATG GAC ATG TTC AGG G-3′ and Cre-R: 5′-CCA GAC CAG GCC AGG TAT CTC T-3′, which produce a 790 bp product following standard PCR. Genotyping for the floxed p53 allele was performed using primers in p53 intron 10, Int10FOR: 5′-AAG GGG TAT GAG GGA CAA GG-3′ and Int10REV: 5′-GAA GAC AGA AAA GGG GAG GG-3′. Using these primers, wildtype p53 alleles produce a 431 bp fragment, while the floxed p53 allele produces a 584 bp fragment following PCR. Successful excision of exons 2–10 by Cre recombinase was verified with PCR using primers Int10REV and Int1FOR (5′-CAC AAA AAC AGG TTA AAC CCA G-3′).

#### p53 allele deletion

Tamoxifen (Sigma Aldrich, St. Louis, MO) was prepared at a concentration of 10 mg/ml in 5% ethanol/95% corn oil (after first suspending in 100% ethanol). To activate the CreERT2 recombinase, CreERT2 allele-containing mice were injected intraperitoneally with 100 µl tamoxifen solution (1 mg tamoxifen per injection) daily for five consecutive days at the ages of 3, 6, and 12 months for aging cohort experiments. To quantitate the relative efficiency of p53 allele deletion in specific tissues of CreERT2-p53^+/F^ and CreERT2-p53^F/F^ mice, genomic DNA was purified as described above from 50 mg of each tissue and 5–10 µg of DNA was digested with 10 units of Bgl II (New England BioLabs, Ipswich, MA) for 16 hours at 37°C. The restricted DNA was subjected to electrophoresis in a 0.8% agarose gel and Southern blotted using standard methods. The blot was hybridized to a ^32^P-labeled mouse p53 exon 11 DNA probe prepared from wildtype genomic DNA with PCR fragments generated from primers Ex11FOR (5-CTA CCT GAA GAC CAA GAA GG-3′) and Ex11REV (5′-TGG AGG ATA TGG ACC CTA TG-3′). After hybridization and washing of the Southern blot followed by autoradiography, the relative intensity of hybridization in the intact p53 allele (20 kb), the excised p53 allele (10 kb) and a p53 pseudogene (2 kb) was quantitated on a GE Molecular Dynamics phosphorimager. Relative efficiencies of excision in tamoxifen-treated CreERT2-p53^+/F^ and CreERT2-p53^F/F^ tissues could be determined after normalizing band intensities to the pseudogene band and to wildtype p53 bands in non-treated control CreERT2-p53^+/F^ and CreERT2-p53^F/F^ tissues.

#### Western blotting

Freshly excised tissue samples were harvested, snap frozen in liquid nitrogen, and stored at -80^°^C until use. Tissues lysates were prepared by homogenization of approximately 50 mg tissue as previously described [36]. For Western blots, 75 µg tissue lysates were loaded per SDS-polyacrylamide gel and analyzed as previously described [36]. Antibodies to p53 (R-19) and p21 (F-5) were from Santa Cruz Biotechnology (Santa Cruz, CA). Actin antibodies were obtained from NeoMarkers (Fremont, CA).

#### Big Blue mutation frequency assay

To determine the effects of tamoxifen on mutation frequencies, wildtype C57BL/6 Big Blue mice from Stratagene were injected daily for five consecutive days with 1 mg of tamoxifen in 100 µl of corn oil. Mice were sacrificed at two or four weeks after the first injection and high molecular weight genomic DNA was prepared from the livers of treated and non-treated animals. The determination of mutation frequencies in the liver DNA of tamoxifen treated and non-treated Big Blue mice was performed according to the manufacturer's guidelines and the methods have previously been described in Hinkal *et al*. [36].

#### Lifespan analyses

All treated and non-treated mice were monitored on a daily basis for morbidity or death as previously described. Severely morbid mice were euthanized. Mice that died overnight and euthanized mice were subjected to gross necropsy and organs and tumor lesions were fixed in 10% neutral buffered formalin, paraffin embedded and hematoxylin and eosin slides prepared in the Veterinary Pathology Core at Baylor College of Medicine. Slides were examined and tumors were classified histopathologically as to type. Survival curve comparisons for each cohort of mice were performed using statistical software for Kaplan-Meier Survival Analysis with Tarone-Ware statistics (SPSS Inc, Chicago). Mortality rates were calculated and compared as described by de Magalhaes *et al*. [37]. Briefly, relative numbers of mouse deaths per tenth of each year were used to calculate the hazard of death with age. These data were compared to other cohort mortality rates on a natural log scale and slopes compared by t-test.

#### Irradiation-induced lymphomagenesis

To assess the effects of p53 allele status on radiation-induced lymphomagenesis, CreERT2-p53^F/F^ mice were treated with tamoxifen for 5 consecutive days as described earlier. 5–7 weeks old mice were subjected to 2.5 Grays whole body ionizing γ-radiation using a Cesium-137 source (MDS Nordion GammaCell 40 Exactor) 2 weeks prior to, concurrent with, 2 weeks after, or 4 weeks after tamoxifen treatment. Mice were then regularly monitored for morbidity or death whence necropsies and tumor-free survival curves were performed as described above.

#### TUNEL assay

For assessing the effects of p53 allele status on IR-induced apoptosis, 3 month old CreERT2-p53^F/F^ mice were injected with tamoxifen (1 mg/day) or vehicle (corn oil) for 5 consecutive days. Three weeks after the first injection, mice were either left untreated or were exposed to 5 Grays whole body γ-irradiation. At 5 h post-irradiation, spleens were harvested and fixed in 10% buffered formalin. Tissues were processed, paraffin embedded, and sections (5 µm) were cut at the Baylor College of Medicine Histology Core. Immunostaining for apoptosis was performed using the In Situ Cell Death Detection Kit, Fluorescein (Roche Applied Science, Indianapolis, IN) as per manufacturer's instructions. Sections were counterstained with DAPI (1 µg/ml) and imaged at 40×magnification under a fluorescence microscope (Zeiss Axioplan2, Carl Zeiss MicroImaging GmbH) using appropriate filters. Fluorescein labeled cells were scored as positive for TUNEL staining. For each tissue section, at least 3 fields were counted and the average percentage of TUNEL positive cells from 3 spleens for every condition was plotted.
